# The impact of biosimilar use on healthcare utilization among new users of etanercept for inflammatory arthritis: a population-based regression discontinuity analysis

**DOI:** 10.1016/j.lana.2025.101058

**Published:** 2025-04-11

**Authors:** Vivienne Yuetong Zhou, Diane Lacaille, Yufei Zheng, Yi Qian, Bohdan Nosyk, Hui Xie

**Affiliations:** a Arthritis Research Canada, 2238 Yukon St #230, Vancouver, BC, V5Y 3P2, Canada; b Faculty of Health Sciences, Simon Fraser University, 8888 University Dr W, Burnaby, BC, V5A 1S6, Canada; c Division of Rheumatology, Department of Medicine, University of British Columbia, 317–2194 Health Sciences Mall, Vancouver, BC, V6T 1Z3, Canada; d Sauder School of Business, University of British Columbia, 2053 Main Mall, Vancouver, BC, V6T 1Z2, Canada; e Center for Health Evaluation & Outcome Sciences, 570–1081 Burrard Street, Vancouver, BC, V6Z 1Y6, Canada

**Keywords:** Health policy, Regression discontinuity, Inflammatory arthritis, Biosimilar, Administrative health data

## Abstract

**Background:**

Epidemiological evidence on biosimilars’ real-world performance is limited. On July 18th, 2017, a biosimilar health policy was implemented in British Columbia (BC), Canada, mandating all patients initiating a new biologic medication to be prescribed a biosimilar (if/when available). Exploiting a policy change as a natural experiment, we assessed the real-world impact of biosimilar use for inflammatory arthritis (IA) on health resource utilization as a surrogate marker of real-world effectiveness and safety.

**Methods:**

Using administrative health data, we identified all incident etanercept users for IA in BC with initiation dates between 2014 and 2020 (n = 3004) [63·6% female; mean (S.D.) age at IA disease diagnosis 52·5 (16·6) years]. Healthcare utilization over three years after initiation was assessed using outcomes including — physician visits (PV), all-cause hospitalizations (ACH), infection-related hospitalizations (IRH), length of hospital stays (LOS), and emergency room visits (ERV). Using regression discontinuity design, we compared healthcare utilization risk in patients initiating etanercept immediately before/after policy-change date, representing the intention-to-treat effect. Additionally, we estimated the complier average causal effect of biosimilar use with instrumental variable (IV) control function method.

**Findings:**

Intention-to-treat analyses showed no significant impact of biosimilar policy implementation on PV, HOSP, IRH, LOS, or ERV, with respective adjusted RRs of 0·96 (95% CI: 0·82–1·12), 0·84 (95% CI: 0·49–1·44), 0·91 (95% CI: 0·21–3·86), 0·94 (95% CI: 0·41–2·15), and 0·91 (95% CI: 0·44–1·88). IV analyses indicated biosimilar use in routine settings did not significantly change healthcare utilization, compared to originator etanercept.

**Interpretation:**

No significant impact of biosimilar policy or actual biosimilar use on healthcare utilization was observed, suggesting equivalent real-world effectiveness and safety of biosimilars to originators and no unintended consequences of the policy change.

**Funding:**

CHIR and 10.13039/501100000038NSERC.

**Research in context**

**Evidence before this study**

Biosimilars' clinical efficacy and safety are well established through randomized clinical trials (RCTs). These studies are the “gold standard” for medical research because they are conducted in highly controlled setting in a carefully selected target population to evaluate treatment efficacy. The term efficacy here is defined as an intervention's effect under “ideal conditions”. After a medication has been adopted for use in the real-world clinical setting, it is also important to evaluate their real-world effectiveness and safety in populations that extend beyond those enrolled in clinical trials. We conducted a literature search on PubMed on observational/interventional studies, systematic reviews, and meta-analyses, published between database inception and Nov 30th, 2024, assessing the effect of biosimilar use for the treatment of inflammatory arthritis using real-world data using the terms (“biosimilar”) AND (“effectiveness” OR “safety”). Existing studies have primarily focused on prevalent users of biologics and evaluated the impact of switching from originators to biosimilars. We found only a few observational studies investigated the impact of biosimilar use among incident biologics users compared to originators. These studies suggest comparable effectiveness/safety between biosimilar and originators. However, a common limitation among these studies is the potential systematic differences between the study and the comparison cohort. Thus, despite a large body of RCTs and observational studies have focused on the evaluation of biosimilars, their full benefit and risk profile remains incompletely defined.

**Added value of this study**

Prevailing body of evidence neglects the need for rigours evaluation of the real-world impact of (1) the implementation of biosimilar policies and (2) the use of biosimilars, while carefully addressing both observed and unobserved confounding. The present study takes advantage of a biosimilar health policy change occurred in British Columbia, Canada as a natural experiment to assess the real-world impact of both policy implementation and biosimilar etanercept use with a regression discontinuity design mimicking population-level real-world RCTs. We investigated healthcare utilization — a surrogate marker for assessing real-world effectiveness and safety of biosimilars in routine clinical practice, which can also reflect the overall impact of biosimilar policy implementation at the health system level. We found no significant impact of policy implementation or actual biosimilar use on healthcare utilization outcomes among incident users of biosimilar vs. originator etanercept for inflammatory arthritis.

**Implications of all the available evidence**

Lack of confidence in biosimilar's effectiveness, safety and interchangeability has been identified as one of the top barriers impairing the adoption of biosimilar therapies. The equivalent real-world effectiveness and safety of biosimilar shown in this study will increase healthcare providers' and patients' confidence in prescribing and taking this important medication. Our findings on no unintended consequences of policy change at the health system level inform decisions in biosimilar policy development and implementation in other jurisdictions.

## Introduction

Inflammatory arthritis (IA) diseases are a diverse group of chronic conditions characterised by synovial inflammation (synovitis), causing pain, swelling, and joint destruction.^1^ If not properly managed, IA diseases can significantly reduce patients’ quality of life and result in disability. The conventional treatment strategies for different forms of IA share similarities, namely, focusing on “treat-to-target” and controlling inflammation to achieve remission.^2^, ^3^, ^4^ The introduction of biologic DMARDs in the late 1990s was a significant advancement in the treatment of IA diseases, marking the beginning of the era of biological treatment.^1^^,^^5^ However, despite their high efficacy, the widespread use of biologics has been limited by their high development and manufacturing cost.^6^

Biosimilars, which are similar versions of brand-name biologics made by different companies once the original drug's patent expires, offer a less expensive alternative, due to the lack of investment in drug development and less extensive testing. To gain regulatory approval, manufacturers must demonstrate that the biosimilar is structurally equivalent to the originator, with similar pharmacokinetics and immunogenicity, and maintains the same efficacy and safety profile, through a more limited clinical trials program than originators.^6^ Etanercept (Enbrel®) is one of the first biologics approved for the treatment of rheumatoid arthritis and is now also approved for juvenile idiopathic arthritis, psoriatic arthritis, axial spondyloarthritis, ankylosing spondylitis, and plaque psoriasis.^7^ In Canada, SB4 (Brenzys®) and GP2015 (Erelzi®) are two approved biosimilar versions of etanercept.

The introduction of biosimilars not only helps expand treatment options for patients but also provides substantial cost savings for the healthcare systems worldwide.^8^ Fair PharmaCare program is British Columbia (BC)'s publicly funded program designed to provide fair access to prescription drug coverage. In BC, the costs for biologic drugs are covered for all patients, as long as they meet specific criteria under PharmaCare Special Authority process.^9^ As biosimilars became available, BC launched the Biosimilars Initiative aiming to increase the uptake of these cost-effective alternatives to enhance PharmaCare's long-term sustainability.^10^ Two policies were implemented: (1) the new start policy and (2) the biosimilar transition policy. This study focuses on the new start policy for etanercept, implemented on July 18th, 2017, mandating all new prescriptions for initiating etanercept treatment use biosimilar versions for cost to be covered by PharmaCare.^11^ This means, PharmaCare no longer covered the cost of originator etanercept for new prescriptions. Consequently, the incident use of etanercept biosimilar has increased drastically after the policy change.

Biosimilars’ clinical efficacy and safety are well established through RCT data.^12^^,^^13^ However, epidemiological evidence on their real-world performance is limited. This new start policy change creates an ideal setting for a quasi-experimental study that utilizes real-world data to assess the impact of biosimilar use in the general population of IA patients and provides insights that might otherwise be challenging or difficult to achieve through the highly controlled RCT environment. In this study, we investigated healthcare utilization — a surrogate marker for assessing real-world effectiveness and safety, which can also reflect the overall impact of biosimilar policy implementation at the health system level. If patients treated with biosimilars show similar healthcare utilization rates to those treated with originators, it suggests equivalent effectiveness and safety outside RCTs.

The objective of this study was to examine the impact of both biosimilar health policy implementation and biosimilar use on healthcare utilization outcomes among incident users of biosimilar vs. originator etanercept for inflammatory arthritis in British Columbia, using a population-based regression discontinuity design.

## Methods

### Study design

In this retrospective cohort study, we employed a regression discontinuity (RD) design. The RD design, first introduced by Thistlethwaite and Campbell (1960), is a quasi-experimental method that provides strong causal impact estimates under certain underlying conditions/assumptions.^14^ The RD design estimates treatment effects in a non-experimental setting where treatment is determined by whether an observed continuous “assignment” (or “running”) variable exceeds a known cut-off point, creating a locally randomized experiment near this threshold. Etanercept initiation date serves as the assignment variable in this study, with cut-off point being policy implementation date — July 18th, 2017. Since treatment assignment is based on date, which is a continuous variable, individuals close to the cut-off (i.e., patients who initiated around July 18th, 2017) are expected to share similar characteristics and prior treatment regimen, including both observed and unobserved confounders.^15^ The exchangeability is the key assumption in the RD design, allowing for unbiased treatment effect estimation, with differences in outcomes between the two groups attributable to treatment rather than confounding.

British Columbia has one of the highest quality administrative data in the world because of its universal health care system that provides comprehensive coverage of healthcare services (Supplementary Appendix S1). These BC administrative databases have been validated and used extensively in population-based health research.^5^^,^^16^, ^17^, ^18^, ^19^, ^20^ We used administrative health data between 2014 and 2023 for the entire population of British Columbia, Canada (5.4 million) accessed through Population Data BC on all provincially funded healthcare services. The databases include data on demographic information from consolidation files based on Medical Services Plan (MSP) registration and premium billing; all physician visits, with one diagnostic code per visit representing the reason for the visit (MSP); all hospitalizations (Discharge Abstract Database [DAD]^21^), which include up to 25 diagnostic codes per hospitalization representing the reason for admission, comorbid diseases or complications during hospitalization; all emergency department visits (National Ambulatory Care Reporting System [NACRS]), and information on death (Vital Statistics^22^); as well as on all medications dispensed to BC residents regardless of source of funding (PharmaNet database). Ethics approval was obtained from the University of British Columbia's behavioural Research Ethics Board (H20-02660). All procedures were compliant with BC's Freedom of Information and Privacy Protection Act.

### Patients

We identified all incident users of etanercept for treatment of IA using drug identification numbers from PharmaNet and primary diagnosis codes in MSP. Incident users of etanercept are defined as without prior prescription of the same agent over 6 months, with prescription start dates between Jan 1st, 2014, and June 30th, 2020, and with brand names: Enbrel, Brenzys, and Erelzi from PharmaNet. Patients could have previous biological agents as long as the agent was not etanercept. IA diseases — rheumatoid arthritis (RA), psoriasis or psoriatic arthritis (Pso/PsA), or ankylosing spondylitis (AS) — were determined using International Classification of Diseases (ICD), 9th version (ICD-9) in MSP and 9th/10th version (ICD-9/ICD-10) in DAD. Specifically, RA was identified by ICD-9 code 714.x and ICD-10 codes M05.x, M06.x, Pso/PsA by ICD-9 code 696.x and ICD-10 code L40.x; and AS by ICD-9 code 720.x and ICD-10 code M45.x. In cases where a patient had multiple disease diagnoses, we selected the disease with diagnosis date closest to the initiation of biologics.

### Outcomes

Healthcare utilization over three year after etanercept initiation was assessed using five outcomes: (1) number of physician visits, (2) number of all-cause hospitalizations, (3) number of infection-related hospitalizations, (4) length of hospital stays, and (5) number of emergency room visits. Physician visit was defined as any outpatient records of physician visits from MSP. Any records of fee-for-service physician visits happened during hospitalization (i.e., inpatient visits by physicians) were excluded. All-cause hospitalization was defined using the “episode of care” criteria, including any contiguous inpatient hospitalizations and same-day surgery visits. If a patient was admitted to one hospital and then transferred to another hospital or to a rehabilitation facility, this would be considered the same episode of care, from which the patient was discharged before readmission. Infection-related hospitalization was defined as any hospitalizations with a diagnostic code for infection in any position in hospitalization data, representing either the reason for admission or an infection occurring during hospitalization,^18^^,^^19^ a case definition that yields a positive predicted value of 0·95.^20^ A total of 58 different types of infections selected a priori by a panel of experts were identified using ICD-9/10 diagnostic codes,^18^ which are available in Supplementary Table S1. Length of hospital stays was calculated as the cumulative number of days during all hospitalization episodes. Emergency room visit was defined as any records of emergency room visits identified from the NACRS databases using the flag EDVisit.

### Follow-up

For each patient, follow-up started at etanercept initiation date and ended at the date of etanercept discontinuation, switching to another type of biologics, death, out-of-province migration or three years of follow-up, whichever came first.

### Covariates assessment

Information on sociodemographic characteristics, comorbidities, and medication use known to be associated with IA conditions and healthcare utilization that were available in our data were identified and included as covariates into the adjusted models. Sociodemographic characteristics included age, sex, socio-economic status (i.e., neighborhood quintile of annual income based on postal code), urban/rural residency and regional health authorities at time of etanercept initiation. Comorbidities and medication use included Romano adaptation of Charlson Comorbidity Index,^23^ diabetes, chronic obstructive pulmonary disease, cardiovascular diseases, renal disease requiring dialysis, methotrexate use, other conventional DMARDs (sulfasalazine, chloroquine, hydroxychloroquine, leflunomide, or cyclosporine) use, glucocorticoid use, and number of different biologic agents used previously, all of which were measured at baseline (within 12 months of etanercept initiation).

### Statistical analysis

To account for differences in individuals' follow-up time due to censoring, we evaluated healthcare utilization rates for each incident etanercept user. The rates were calculated as the total number of outcomes occurring during each individual's follow-up time divided by their respective follow-up time. With the RD design, we conducted two analyses of individual patients' health utilization outcomes: (1) an intention-to-treat (ITT) analysis and (2) a complier average causal effect (CACE) analysis. For ITT analysis, we compared risks of healthcare utilization in patients who initiated etanercept immediately before and immediately after policy change date (i.e., cut-off point), regardless of whether a biosimilar or originator was used, to assess the effect of policy implementation. We first plotted kernel-weighted local linear regression (LLR) curves of mean event rates over calendar date of etanercept initiation.^24^ The optimal bandwidths (h) around the cut-off for each outcome were obtained using the Imbens-Kalyanaram data-driven bandwidth algorithm.^25^ These bandwidths represent the largest interval around cut-off where the relationship between time and outcome is nearly linear^25^ and will be utilized in obtaining the ITT effect next. Given outcomes are event counts in nature, we focused on rate ratio estimation and used the quasi-Poisson count regression model for over-dispersed count data with a log link function and the log of follow-up time as an offset. Our quasi-Poisson model included two separate curves of etanercept initiation time (measured as days from policy change date) for periods before and after policy change and an indicator variable for policy change to capture the discontinuity in the outcome at policy implementation (see Supplementary Appendix S2 for a more detailed model description). To reduce noise and increase precision^26^ and account for potential changes in cohort composition,^27^ we adjusted for measured baseline covariates into our model.

In the second analysis, we estimated the CACE effect of actual biosimilar use on healthcare utilization via quasi-Poisson regression with the instrumental variable (IV) control function estimator method (Supplementary Appendix S2), where the IV is an indicator variable for whether a patient started etanercept before [0] or after [1] policy change date. This involved firstly regressing biosimilar use (i.e., an indicator variable for whether a patient was treated by biosimilar [1] or originator [0]) on the IV and baseline covariates and obtaining the residual from this first-stage regression. Then, the second-stage quasi-Poisson model included two separate curves of etanercept initiation time for originator and biosimilar users, respectively, an indicator variable for biosimilar use (to capture the outcome difference for biosimilar vs. originator use at policy implementation), baseline covariates, and the residual of first-stage regression as the control function to control for potential selection bias due to unobserved confounders of biosimilar use. The coefficients obtained from the second-stage regression were taken as the control function estimates.^28^ The CACE analysis assessed the impact of biosimilar use on healthcare utilization among policy compliers, specifically those induced to take up the treatment according to policy implementation. Statistical analyses were performed in SAS (Version-9.4), R (Version-4.3.1), and STATA (Version-16.1).

### Sensitivity analysis

We conducted several sensitivity analyses. In the first sensitivity analysis (Supplementary Table S2), we implemented a non-parametric LLR approach to estimate both the ITT and CACE effects as risk differences using the same bandwidths as our primary analysis.^24^ Then, to check the robustness of our results to different bandwidths, we repeated our analysis with data-driven bandwidths obtained via the mean square error (MSE)-optimal bandwidths algorithm.^29^ Results obtained from the parametric quasi-Poisson regression approach and the non-parametric LLR approach (both with new bandwidths) were reported in Supplementary Tables S3 and S4, respectively. We conducted a separate intention-to-treat (ITT) analysis among adalimumab initiators with the same setup as our main analysis (e.g., cut-off set at the etanercept policy change date) to assess any confounding by unobserved time-specific events (Supplementary Table S5). Although COVID pandemic might more likely affect routine healthcare utilization, less urgent healthcare utilization such as physician visits were done virtually in BC to minimize the impacts of COVID. Nonetheless, to explicitly account for the potential impact of COVID-19, we conducted a sensitivity analysis excluding patients initiating etanercept on and after Mar 1, 2019, and then followed the patients for 1-year so that follow up period is the same for all patients and has no overlap with COVID pandemic (Supplementary Tables S6 and S7).

### Assumption assessment

To obtain the ITT effect of policy implementation, the main underlying assumption is the exchangeability assumption^15^: if the treatment assignment is random, individuals immediately above/below the threshold would have a similar distribution of both measured and unmeasured baseline characteristics, resulting in exchangeability. We discussed and performed falsification tests to assess the key underlying assumptions in Supplementary Appendix S3. To obtain the CACE effect of actual biosimilar use, we employed RD design in combination of an IV approach (i.e., fuzzy RD) and compared the outcomes of policy compliers who used biosimilars versus who used originators close to cut-off. We discussed further details of our IV model (demonstrated with a causal diagram) and assessments of assumptions (i.e., relevance, exchangeability, exclusion restriction, and monotonicity) in Supplementary Appendix S4 and Supplementary Figures S1–S5.

### Role of the funding source

The funder of the study had no role in study design, data collection, data analysis, data interpretation, or writing of the report. We are not paid to write this article by a pharmaceutical company or other agency. The authors were not precluded from accessing data in the study, and they accept responsibility to submit for publication.

## Results

**Fig. 1:**
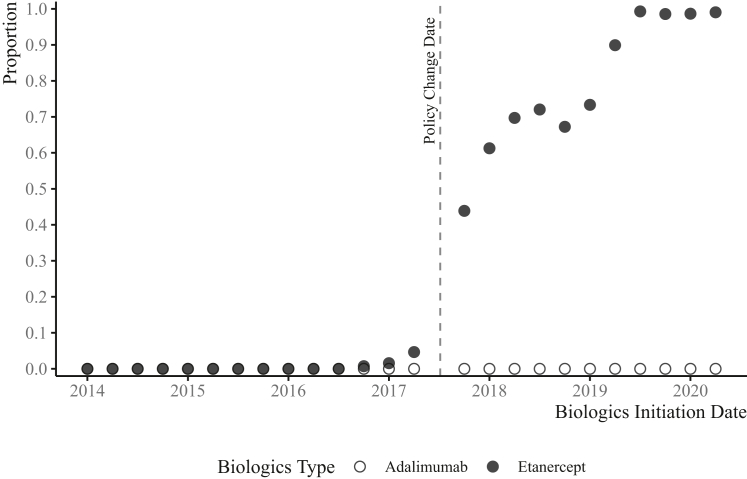
Proportion of biosimilar use. Note: Biologics incident users were divided into quarterly cohorts (4 per year) according to their biologics initiation date (except for the datapoints appear immediately before and after policy change date). The datapoint appears immediately before policy change date (i.e., grey dashed line) included biologics incident users whose initiation date were between April 1st, 2017, and July 17th, 2017, inclusive. The datapoint appears immediately after policy change date (i.e., grey dashed line) included biologics incident users whose initiation date were between July 18th, 2017, and Dec 31st, 2017, inclusive. The proportion of biosimilar use was calculated as: the number of biosimilar users in each cohort divided by the total number of biologics users (both biosimilar and originator users) in each cohort.

We identified 1626 incident users of etanercept with treatment initiation in the pre-policy period between Jan 1st, 2014 and July 17th, 2017 [63·5% female; mean (S.D.) age at diagnosis 51·6 (16·4) years] and 1378 incident users of etanercept in the post-period between July 18th, 2017 and June 30th, 2020 [63·7% female; mean (S.D.) age at diagnosis 53·6 (16·7) years]. Baseline characteristics of incident etanercept users in pre/post-policy periods are reported in Table 1. Covariates balance tests conclude all measured characteristics are similar for patients who started etanercept around the cut-off (Supplementary Figures S2–S4), which supports a locally randomized experiment around the cut-off. The proportion of biosimilar users increased significantly from 0·06% (9 out of 1626) to 78·3% (1079 out of 1378) from pre-policy to post-policy period. Fig. 1 shows the proportion of biosimilar use among all incident etanercept users over time with a significant discontinuity observed at policy change (P < 0·0001). The imperfect compliance pattern in the proportion change, where few etanercept users initiated treatment with biosimilar before policy change, and not all etanercept users initiated treatment with biosimilar after policy change, requires using a fuzzy RD design approach to evaluate the treatment effect.^14^^,^^15^ The incomplete compliance post policy (i.e., initiating originator despite the policy change) is likely related to some users having coverage through private insurance. Incident adalimumab users in this figure serve as a comparison group, with no biosimilar adalimumab available throughout the study period, resulting in the proportion of biosimilar use remaining at zero.

**Table 1:** Baseline characteristics for incident etanercept users for inflammatory arthritis in BC, Canada, with etanercept initiation dates between 2014 and 2020.

	Pre-policy period	Post-policy period	P-value^a^
	(Jan 1, 2014–July 17, 2017)	(July 18, 2017–June 30, 2020)	
**Characteristics**			
N	**1626**	**1378**	
Biosimilar, N (% of biosimilar use)	**9 (9/1626 = 0·6%)**	**1079 (1079/1378 = 78·3%)**	<0·0001
Age at initiation (years), mean (S.D.)	51·6 (16·4)	53·6 (16·7)	0·0008
Female, N (%)	1033 (63·5%)	878 (63·7%)	0·92
Quintile of Annual Income Per Person Equivalent, N (%)			0·09
*1 (Lowest)*	316 (19·4%)	293 (21·3%)	
*2 (Low)*	327 (20·1%)	294 (21·3%)	
*3 (Medium)*	337 (20·7%)	272 (19·7%)	
*4 (High)*	337 (20·7%)	280 (20·3%)	
*5 (Highest)*	309 (19·0%)	239 (17·3%)	
Rural, N (%)	257 (15·8%)	263 (19·1%)	0·019
Health Authority, N (%)			0·97
*Interior*	418 (25·7%)	367 (26·6%)	
*Fraser*	551 (33·9%)	436 (31·6%)	
*Vancouver Coastal*	304 (18·7%)	280 (20·3%)	
*Vancouver Island*	260 (16·0%)	220 (16·0%)	
*Northern*	93 (5·7%)	75 (5·4%)	
**Comorbidities**			
CCI, mean (S.D.)	0·7 (0·8)	0·8 (0·9)	0·0002
COPD, %	12·2%	13·1%	0·46
DB, %	3·4%	3·3%	0·86
Renal Disease requiring dialysis, %	3·2%	3·7%	0·45
CVDs, %	5·0%	5·2%	0·76
**Medication use**			
Number of types of biologics agent used previously, mean (S.D.)	0·4 (0·7)	0·5 (0·8)	0·004
MTX, %	37·1%	38·7%	0·39
Other csDMARDs, %	42·4%	42·9%	0·78
Glucocorticoids, %	27·8%	28·2%	0·79
**Outcomes**			
Total follow-up, person-years	2784	2045	
Number of physician visits	151,465	105,405	
Number of all-cause hospitalizations	893	689	
Number of infection-related hospitalizations	55	43	
Total length of hospital stays	3393	2185	
Number of emergency room visits	1334	943	

Abbreviations: CCI, Charlson Comorbidity Index; COPD, chronic obstructive pulmonary diseases; csDMARDs, conventional disease-modifying anti-rheumatic drugs; CVD, cardiovascular diseases; DB, diabetes; MTX, methotrexate; S.D., standard deviation.

a P-values obtained from two-sample t test (pre-period vs post-period).

**Fig. 2:**
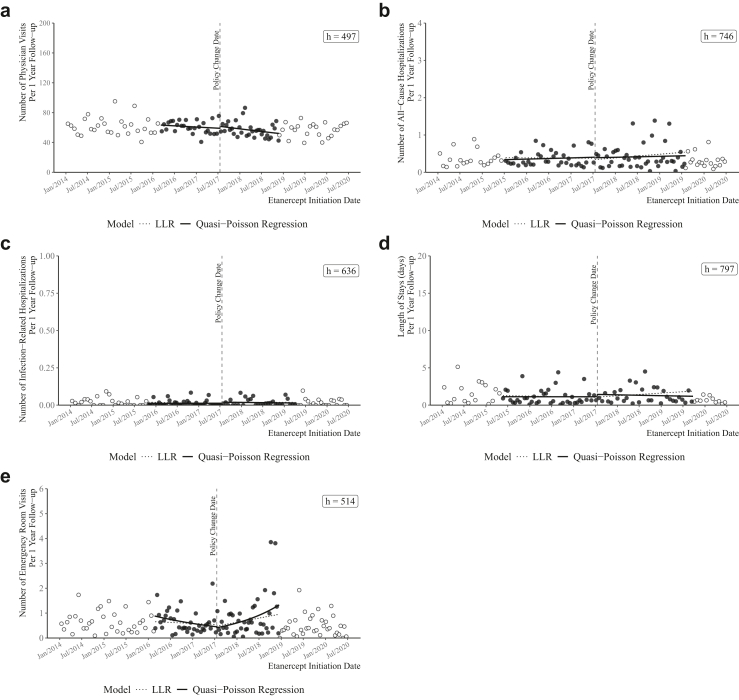
Healthcare utilization outcomes by etanercept initiation date. (a) Physician Visits; (b) All-Cause Hospitalizations; (c) Infection-Related Hospitalizations; (d) Length of Hospital Stays; (e) Emergency Room Visits. Note: The black solid line shows estimates obtained from quasi-Poisson Regression (i.e., primary results). The black dotted line shows estimates obtained from local linear regression (LLR),^24^ which serve as the comparison to the quasi-Poisson estimates. The dots are binned averages of each outcome, with black dots representing data points that fall within the data-driven optimal bandwidths (h).

Fig. 2 depicts the rates of individual utilization outcomes among incident etanercept users over the study period. No significant changes in the rates of any of the five utilization outcomes were observed at the policy change date (i.e., cut-off). Table 2 and Table 3 show the results from the ITT and CACE analysis, respectively. Our ITT analysis showed that, the unadjusted and adjusted ratios of PV rates (i.e., expected numbers of physician visits per 1 follow-up year) at cut-off were 1·04 (95% CI: 0·87–1·26) and 0·96 (95% CI: 0·82–1·12), respectively, indicating no significant impact of policy change on PV risk among incident etanercept users. For the CACE effect, the unadjusted and adjusted ratios of PV rates at cut-off were 0·93 (95% CI: 0·73–1·20) and 0·84 (95% CI: 0·66–1·06), suggesting that no significant impact of biosimilar use on PV risk was observed among policy compliers (i.e., those induced to take up biosimilar after policy change).

**Table 2:** The intention-to-treat effect of policy implementation on healthcare utilization outcomes among incident etanercept users.

Outcome	Parameter	Pre-policy	Post-policy	Unadjusted effect estimates	Adjusted^a^ effect estimates	Bandwidths^b^
		(95% CI)	(95% CI)	(95% CI) P-value	(95% CI) P-value	
PV rate	ITT	59·00 (52·08–66·83)	61·63 (53·88–70·49)	1·04 (0·87–1·26) 0·64	0·96 (0·82–1·12) 0·58	497
ACH rate	ITT	0·4 (0·26–0·63)	0·39 (0·28–0·55)	0·97 (0·55–1·69) 0·90	0·84 (0·49–1·44) 0·52	746
IRH rate	ITT	0·01 (0·005–0·04)	0·02 (0·01–0·05)	1·55 (0·39–6·18) 0·54	0·91 (0·21–3·86) 0·90	636
LOS	ITT	1·13 (0·59–2·16)	1·44 (0·86–2·42)	1·28 (0·55–2·93) 0·57	0·94 (0·41–2·15) 0·89	797
ERV rate	ITT	0·45 (0·28–0·73)	0·41 (0·23–0·74)	0·92 (0·43–1·96) 0·83	0·91 (0·44–1·88) 0·80	514

Estimates obtained from quasi-Poisson regression with optimal bandwidths around the cut-off obtained via Imbens and Kalyanaraman bandwidths selector.^25^ Bandwidths represent days or distance from July 18, 2017. Rates were defined as number of outcomes per 1 follow-up year. ITT effect estimates were in rate ratios. Confidence intervals (CIs) obtained using critical value = 1·96.

Abbreviations: ACH, all-cause hospitalization; CI, confidence interval; ERV, emergency room visit; IRH, infection-related hospitalization; ITT, intention-to-treat; LOS, length of hospital stays; PV, physician visit.

a Adjusted baseline covariates include sociodemographic characteristics, comorbidities, and medication use.

b Bandwidths used for estimating both the unadjusted and adjusted effects.

**Table 3:** The complier average causal effect of biosimilar use on healthcare utilization outcomes among incident etanercept users.

Outcome	Parameter	Unadjusted effect estimates	Adjusted^a^ effect estimates
		(95% CI) P-value	(95% CI) P-value
PV rate	CACE	0·93 (0·73–1·20) 0·58	0·84 (0·66–1·06) 0·13
ACH Rate	CACE	0·97 (0·53–1·75) 0·91	1·01 (0·53–1·92) 0·97
IRH rate	CACE	0·98 (0·09–10·54) 0·99	0·89 (0·03–23·35) 0·94
LOS	CACE	1·03 (0·53–1·99) 0·93	0·92 (0·46–1·82) 0·80
ERV rate	CACE	0·88 (0·46–1·69) 0·71	0·94 (0·47–1·88) 0·86

Estimates obtained using quasi-Poisson regression with instrumental variable (IV) control function method. Rates were defined as number of outcomes per 1 follow-up year. CACE estimates were in rate ratios. Confidence intervals (CIs) obtained using critical value = 1·96.

Abbreviations: ACH, all-cause hospitalization; CACE, complier average causal effect; CI, confidence interval; ERV, emergency room visit; IRH, infection-related hospitalization; LOS, length of hospital stays; PV, physician visit.

a Adjusted baseline covariates include sociodemographic characteristics, comorbidities, and medication use.

For all-cause hospitalizations (ACH), ITT analysis yielded unadjusted and adjusted ratios of ACH rates at cut-off of 0·97 (95% CI: 0·55–1·69) and 0·84 (95% CI: 0·49–1·44), suggesting no significant impact of policy change on hospitalization risk among incident etanercept users was observed. The CACE analysis similarly indicated no observed impact from biosimilar use on hospitalization risk among policy compliers, with ratios of 0·97 (95% CI: 0·53–1·75) and 1·01 (95% CI: 0·53–1·92).

For infection-related hospitalizations (IRH), ITT analysis showed unadjusted and adjusted IRH ratios of 1·55 (95% CI: 0·39–6·18) and 0·91 (95% CI: 0·21–3·86). The CACE analysis yielded similar results, with unadjusted and adjusted IRH ratios of 0·98 (95% CI: 0·09–10·54) and 0·89 (95% CI: 0·03–23·35). Given the rarity of IRH events, both ITT and CACE estimates have very wide confidence intervals. Therefore, it is possible the observed lack of significant impact from policy implementation and biosimilar use on risk of IRHs was due to low statistical power.

No evidence of significant impact of policy implementation or biosimilar use on both length of stay (LOS) and emergency room visits (ERV) was observed. Specifically, ITT analysis showed unadjusted and adjusted ratios of LOS at cut-off of 1·28 (95% CI: 0·55–2·93) and 0·94 (95% CI: 0·41–2·15), and unadjusted and adjusted ratios of ERV rates at cut-off of 0·92 (95% CI: 0·43–1·96) and 0·91 (95% CI: 0·44–1·88), respectively. CACE analysis showed similar findings of 1·03 (95% CI: 0·53–1·99) and 0·92 (95% CI: 0·46–1·82) for LOS and 0·88 (95% CI: 0·46–1·69) and 0·94 (95% CI: 0·47–1·88) for ERV, respectively.

Sensitivity analysis using LLR (Supplementary Table S2) to estimate ITT and CACE effects on the scale of rate differences yields the same conclusion as primary analysis of rate ratios. Sensitivity analyses using different bandwidths also showed similar results (Supplementary Tables S3 and S4). This suggests that our findings are robust across different analytical approaches (i.e., parametric quasi-Poisson regression vs. non-parametric LLR), effect measures (i.e., rate ratio vs. rate difference), and choice of bandwidths algorithms (i.e., IK vs. MSE-optimal algorithm). ITT analysis among adalimumab initiators showed no significant differences in healthcare utilization outcome at etanercept policy cut-off (Supplementary Table S5), confirming that our findings are unlikely to be confounded by any unobserved time-specific events. Results from the sensitivity analyses accounting for the potential impact of the COVID-19 pandemic on healthcare utilization showed results consistent with the study's overall findings on the equivalence of biosimilars to originators (Supplementary Tables S6 and S7).

## Discussion

The effectiveness and safety of biosimilars have been well demonstrated in RCTs, but there is limited clinical or epidemiological data available on the comparison of healthcare utilization between biosimilars and originators incident users. The biosimilar policy change occurred in BC creates an ideal setting for a quasi-experimental study using real-world data to assess biosimilar's impact in an unselected population. In this population-based cohort study, we examined the impact of biosimilar policy implementation and biosimilar use on healthcare utilization outcomes among incident users of biosimilar vs. originator etanercept for IA, using a population-based regression discontinuity design. No significant impact of biosimilar policy implementation or actual biosimilar use was observed among incident etanercept users.

Existing observational studies have primarily focused on prevalent users of biologics and evaluated the impact of switching from originators to biosimilars. A Danish study^30^ using the DanBiO registry explored the impact of a mandatory non-medical switch from originator to biosimilar etanercept and found no significant changes in overall use and costs of healthcare services in 1620 Danish patients, where patients served as their own controls before and after switch. Another study^31^ assessed the impact of a non-medical recommendation of switching in a region of Italy. By comparing patients treated with infliximab on January 1st, 2013 (no biosimilar available) to those on January 1st, 2016 (biosimilar available), the study found no relevant changes in the clinical outcomes following biosimilar introduction. In Canada, a study^32^ investigated healthcare use following the mandatory biosimilar switching policy in BC. The study compared policy vs. historical cohorts of etanercept users and did not find permanent unintended changes in healthcare use during 1-year follow-up, suggesting that switching to biosimilar etanercept had minimal impacts on patient health. Common limitations of these studies include potential confounding by time effects and prevalent user bias.^33^

A few observational studies investigated the impact of biosimilar use among incident biologics users compared to originators.^34^, ^35^, ^36^, ^37^ These studies mainly focused on the efficacy and safety aspects and discovered comparable effectiveness or safety between biosimilars and originators. A common limitation among these studies is the potential systematic differences between the biosimilar and originator cohort. For example, a UK study focusing on biologic-naïve patients (n = 1806) evaluated the effectiveness of etanercept originator vs. biosimilar^37^ and found similar drug survival and disease activity between two cohorts after 6/12 months of therapy. However, as mentioned by the authors, potential biases of the study include any systematic differences between the treatment and control cohort in terms of patients’ characteristics. The treatment cohort included patients who had started using biosimilar more recently (since 2016), while the control cohort started on originators since 2010. While propensity score matching addressed observed confounding in the study, unobserved confounding may still exist.

Our study differs from existing studies in a number of ways and adds important new knowledge to the emerging pool of cause-and-effect studies of biosimilar use. Firstly, to our knowledge, this study is the first application of an RD design to investigate the impact of biosimilar use. In this study, we take advantage of a health policy change as a natural experiment, creating a locally randomized experiment around policy cut-off that accounts for observed and unobserved confounders. We use etanercept initiation date as our running variable with policy cut-off point determining biosimilar use eligibility. Unlike the simple RD in time design, we rely on asymptotics in *N* (i.e., number of cross-sectional units) rather than in *T* (i.e., number of time-points/periods).^27^ The RD analysis is expected to be robust to the occurrence of other time-specific events (e.g., COVID pandemic) because the RD analysis is driven primarily by observations locally around the cut-off point: data points near the policy change time and remote from the start of other events (e.g., COVID pandemic) are weighted more in the analysis. The two sensitivity analyses—one that uses Adalimumab as a control group to account for differences in care over that time period for reasons other than the health policy, and another that excludes the follow-up period overlapping with COVID pandemic—further confirm the robustness of the RD analysis. Secondly, our study utilizes a well-defined population-based incident cohort of etanercept users, protecting against potential selection bias^33^ that could result from prevalent cohort which was predominantly used in prior studies. For example, there might be an increasing use of healthcare services in patients that had previously been treated with etanercept for a longer time-period.^30^ Using incident etanercept users also reduces the potential nocebo effect, as these individuals have minimal prior exposure, reducing potential biases or negative expectations about the effectiveness and safety of biosimilar compared to prevalent users. In addition, we conducted multiple sensitivity analyses to verify the robustness of our results under different model specifications and bandwidths choices.

Some limitations exist in this study. The validity of RD design depends closely on key underlying assumptions. We have empirically tested for violations of some assumptions; however, not all can be empirically proven.^38^ Instead, they can be supported by subject knowledge and/or falsified through extensive testing. Another limitation is that the CACE effect reported only reflects biosimilar's local average treatment effect around cut-off. As such, results were only generalizable to patients who initiated biosimilar etanercept around policy implementation and cannot be assumed to hold for those with significantly different characteristics or circumstances from those at policy cut-off. For the less common outcomes such as infection-related hospitalization, the wide confidence intervals reflect high uncertainty in their estimates. Future studies with extended follow-up period or larger sample size are warranted to improve precision and statistical power to detect small but clinically important effects. The lack of data on direct measures of disease activity and severity in our administrative data also poses some important limitations. In our study, we investigated healthcare utilization outcomes to reflect the impact of policy implementation at the healthcare system level. These outcomes also act as surrogates for the effectiveness and safety of the biosimilar treatment. However, it is important to note that changes in outcomes related to disability and in outcomes related to healthcare services not provincially funded (e.g., physiotherapy, occupational therapy, massage therapy, acupuncture, and naturopathy) are not captured. Additionally, the time frame of the study and the recency of this biosimilar policy implementation do not allow for the evaluation of long-term outcomes such as joint replacement surgery (i.e., a key long-term outcome for patients with poor disease control). As such, the effectiveness of biosimilar as compared to originators on these metrics requires further study. This study focuses on the overall population impacts of biosimilar use on healthcare utilization outcome. The administrative data also lacks information on gender and race/ethnicity, preventing investigation of any differential effects of biosimilar policy or usage across different population subgroups. Future research can assess whether the impact of biosimilar use varies across diverse population subgroups.

In conclusion, our study takes advantage of a biosimilar health policy change as a natural experiment to assess the impact of policy implementation and biosimilar use using a regression discontinuity design. No significant impact of policy implementation and biosimilar use on healthcare utilization among incident etanercept users was observed, suggesting equivalent real-world effectiveness and safety of biosimilars to originators and no unintended consequence of policy implementation.

## Contributors

VYZ: conceptualization, methodology, data analysis, writing—original draft, review & editing; DL: funding acquisition, methodology, clinical interpretations of the results, and writing—review & editing; YZ: data management and analysis; YQ and BN: methodology and writing—review & editing; HX: supervision, funding acquisition, conceptualization, methodology, and writing—review & editing. VYZ and YZ directly accessed and verified the underlying data. VYZ and HX were responsible for the decision to submit the manuscript.

## Data sharing statement

All the data are made available via Population Data BC (https://www.popdata.bc.ca/). Access to data provided by the Data Stewards is subject to approval but can be requested for research projects through the Data Stewards or their designated service providers. The following data sets were used in this study: consolidation, hospital separations (DAD), MSP Practitioner File, NACRS, PharmaNet, and VS–deaths. You can find further information regarding these data sets by visiting the PopData project webpage at: https://my.popdata.bc.ca/project_listings/21-055/collection_approval_dates. All inferences, opinions, and conclusions drawn in this publication are those of the author(s), and do not reflect the opinions or policies of the Data Steward(s).

## Declaration of interests

The authors declare no conflicts of interest.

## References

[1] Pisetsky D.S., Ward M.M. (2012). Advances in the treatment of inflammatory arthritis. Best Pract Res Clin Rheumatol.

[2] Fraenkel L., Bathon J.M., England B.R. (2021). 2021 American College of Rheumatology Guideline for the treatment of rheumatoid arthritis. Arthritis Rheumatol.

[3] Singh J.A., Guyatt G., Ogdie A. (2019). 2018 American College of Rheumatology/National Psoriasis Foundation Guideline for the treatment of psoriatic arthritis. Arthritis Care Res.

[4] Ward M.M., Deodhar A., Gensler L.S. (2019). 2019 Update of the American College of Rheumatology/Spondylitis Association of America/Spondyloarthritis Research and Treatment Network recommendations for the treatment of ankylosing spondylitis and nonradiographic axial spondyloarthritis. Arthritis Rheumatol.

[5] Zhou V.Y., Lacaille D., Lu N. (2022). Has the incidence of total joint arthroplasty in rheumatoid arthritis decreased in the era of biologics use? A population-based cohort study. Rheumatology.

[6] Smolen J.S., Goncalves J., Quinn M., Benedetti F., Lee J.Y. (2019). Era of biosimilars in rheumatology: reshaping the healthcare environment. RMD Open.

[7] Hassett B., Singh E., Mahgoub E., O'Brien J., Vicik S.M., Fitzpatrick B. (2018). Manufacturing history of etanercept (Enbrel®): consistency of product quality through major process revisions. MAbs.

[8] Kvien T.K., Patel K., Strand V. (2022). The cost savings of biosimilars can help increase patient access and lift the financial burden of health care systems. Semin Arthritis Rheum.

[9] British Columbia Ministry of Health Special authority. https://www2.gov.bc.ca/gov/content/health/practitioner-professional-resources/pharmacare/prescribers/special-authority#.

[10] British Columbia Ministry of Health Biosimilar initiative for health professionals. https://www2.gov.bc.ca/gov/content/health/practitioner-professional-resources/pharmacare/prescribers/biosimilars-initiative-health-professionals.

[11] British Columbia Ministry of Health BC PharmaCare Newsletter. https://www2.gov.bc.ca/assets/gov/health/health-drug-coverage/pharmacare/newsletters/pharmacare_newsletter_july_2017.pdf.

[12] Komaki Y., Yamada A., Komaki F. (2017). Efficacy, safety and pharmacokinetics of biosimilars of anti-tumor necrosis factor-α agents in rheumatic diseases; a systematic review and meta-analysis. J Autoimmun.

[13] Andrade A.M., da Motta Girardi J., da Silva E.T., Barbosa J.R., Pereira D.C.R. (2024). Efficacy, safety, and immunogenicity of biosimilars compared with the biologic etanercept in patients with rheumatoid arthritis: a systematic review and meta-analysis. Syst Rev.

[14] Thistlethwaite D.L., Campbell D.T. (1960). Regression-discontinuity analysis: an alternative to the ex post facto experiment. J Educ Psychol.

[15] Moscoe E., Bor J., Bärnighausen T. (2015). Regression discontinuity designs are underutilized in medicine, epidemiology, and public health: a review of current and best practice. J Clin Epidemiol.

[16] Chang J., Rogers P., Lacaille D. (2011). Can American college of rheumatology criteria for rheumatoid arthritis Be assessed using self-report data? Comparison of self-reported data with chart review. Arthritis Rheum.

[17] Aviña-Zubieta J.A., Chan J., Vera M.D., Sayre E.C., Choi H., Esdaile J. (2019). Risk of venous thromboembolism in ankylosing spondylitis: a general population-based study. Ann Rheum Dis.

[18] Lacaille D., Guh D.P., Abrahamowicz M., Anis A.H., Esdaile J.M. (2008). Use of nonbiologic disease-modifying antirheumatic drugs and risk of infection in patients with rheumatoid arthritis. Arthritis Rheum.

[19] Zhou V.Y., Lacaille D., Lu N. (2023). Risk of severe infections after the introduction of biologic DMARDs in people with newly diagnosed rheumatoid arthritis: a population-based interrupted time-series analysis. Rheumatology.

[20] Barber C., Lacaille D., Fortin P.R. (2013). Systematic review of validation studies of the use of administrative data to identify serious infections. Arthritis Care Res.

[21] Canadian Institute for Health Information [creator] (2022). Discharge abstract database (hospital separations). V2. Population data BC [publisher]. Data extract. MOH. http://www.popdata.bc.ca/data.

[22] BC Vital Statistics Agency [creator] (2022). Vital Statistics death. V2. Population data BC [publisher]. Data extract BC vital statistics agency (2022). http://www.popdata.bc.ca/data.

[23] Romano P.S., Roos L.L., Jollis J.G. (1993). Adapting a clinical comorbidity index for use with ICD-9-CM administrative data: differing perspectives. J Clin Epidemiol.

[24] Calonico S., Cattaneo M.D., Titiunik R. (2014). Robust data-driven inference in the regression-discontinuity design. STATA J.

[25] Imbens G., Kalyanaraman K. (2012). Optimal bandwidth choice for the regression discontinuity estimator. Rev Econ Stud.

[26] Lee D.S., Lemieux T. (2010). Regression discontinuity designs in economics. J Econ Lit.

[27] Hausman C., Rapson D. (2017). Regression discontinuity in time: considerations for empirical applications. https://papers.ssrn.com/abstract=3007473.

[28] Guo Z., Small D.S. (2016). Control function instrumental variable estimation of nonlinear causal effect models. J Mach Learn Res.

[29] Calonico S., Cattaneo M.D., Titiunik R. (2014). Robust nonparametric confidence intervals for regression-discontinuity designs. Econometrica.

[30] Glintborg B., Ibsen R., Bilbo R.E.Q., Hetland M.L., Kjellberg J. (2019). Does a mandatory non-medical switch from originator to biosimilar etanercept lead to increase in healthcare use and costs? A Danish register-based study of patients with inflammatory arthritis. RMD Open.

[31] Convertino I., Lucenteforte E., Gini R. (2021). Utilisation patterns and clinical impact of the introduction of infliximab-biosimilar in Tuscany, Italy: real world evidence following the recommendation of switching for non-medical reasons. Clin Exp Rheumatol.

[32] Fisher A., Kim J.D., Carney G., Dormuth C. (2022). Rapid monitoring of health services use following a policy to switch patients from originator to biosimilar etanercept—a cohort study in British Columbia. BMC Rheumatology.

[33] Choi H.K., Nguyen U.-S., Niu J., Danaei G., Zhang Y. (2014). Selection bias in rheumatic disease research. Nat Rev Rheumatol.

[34] Lindström U., Glintborg B., Giuseppe D.D. (2019). Treatment retention of infliximab and etanercept originators versus their corresponding biosimilars: nordic collaborative observational study of 2334 biologics naïve patients with spondyloarthritis. RMD Open.

[35] Thiele F., Klein A., Hospach A. (2021). Efficacy and safety of etanercept biosimilars compared with the originator for treatment of juvenile arthritis: a prospective observational study. ACR Open Rheumatol.

[36] Pinto A.S., Cunha M.M., Pinheiro F. (2022). Effectiveness and safety of original and biosimilar etanercept (Enbrel® vs Benepali®) in bDMARD-naïve patients in a real-world cohort of Portugal. ARP Rheumatol.

[37] Kearsley-Fleet L., Rokad A., Tsoi M.-F. (2023). Etanercept originator versus etanercept biosimilar for the treatment of rheumatoid arthritis as a first biologic: results from the BSRBR-RA. Rheumatology.

[38] Swanson S.A., Hernán M.A. (2013). Commentary: how to report instrumental variable analyses (suggestions welcome). Epidemiology.

