# Supplementary material for: The impact of biosimilar use on healthcare utilization among new users of etanercept for inflammatory arthritis: a population-based regression discontinuity analysis
Source: Lancet Reg Health Am. 2025 Apr 11;45:101058. doi: 10.1016/j.lana.2025.101058 (PMC12017995; doi:10.1016/j.lana.2025.101058)
Supplement: Supplementary Figures and Tables [file mmc1.pdf]

SUPPLEMENTARY MATERIALS: The impact of biosimilar use on healthcare utilization among new users of etanercept for inflammatory arthritis: a population-based regression discontinuity analysis

Table of Contents

Supplementary Appendix S1. The BC databases. 3

Supplementary Table S1. ICD-9 and ICD-10 codes for infection. 4

Supplementary Appendix S2. The ITT and CACE models. 6

Supplementary Table S2. The intention-to-treat effect of policy implementation and complier average causal effect of biosimilar use on healthcare utilization outcomes via local linear regression. 7

Supplementary Table S3. The intention-to-treat effect of policy implementation on healthcare utilization outcomes with MSE-optimal bandwidths via quasi-Poisson Regression. 8

Supplementary Table S4. The intention-to-treat effect of policy implementation and complier average causal effect of biosimilar use on healthcare utilization outcomes with MSE-optimal bandwidths via local linear regression. 9

Supplementary Table S5. The intention-to-treat effect of policy implementation on healthcare utilization outcomes among adalimumab initiators: A robustness check. 10

Supplementary Table S6. The intention-to-treat effect of policy implementation on healthcare utilization outcomes excluding data overlapping with the COVID-19 period. 11

Supplementary Table S7. The complier average causal effect of biosimilar use on healthcare utilization outcomes excluding data overlapping with the COVID-19 period. 12

Supplementary Appendix S3. Assessing underlying assumptions in our ITT Analysis (A sharp RD design). 13

Supplementary Appendix S4. Assessing underlying assumptions in our CACE Analysis (A fuzzy RD design with IV analysis). 14

Supplementary Figure S1. Density plot of the running variable. 16

Supplementary Figure S2. Covariates balance check (demographics). 17

Supplementary Figure S3. Covariates balance check (comorbidities). 18

Supplementary Figure S4. Covariates balance check (medication use). 19

Supplementary Figure S5. A causal diagram demonstrating our fuzzy RD with IV estimation.................................20

References 21

Supplementary Appendix S1. The BC databases.

Complete health information for physician visits, medications dispensed, and hospitalizations were obtained from administrative databases of the Ministry of Health, through Population Data BC. Within this public healthcare system, all persons are guaranteed universal coverage for physician visits, hospital, and medical services.

1) Pharmanet Database (01/1996 onwards) provides information on all prescriptions dispensed by pharmacies in BC, regardless of source of funding.[1]

2) Medical Service Plan File (01/1990 onwards) contains physician claims data used for reimbursement of physician visits, under fee-for-service billings. Each claim contains a single diagnosis representing the reason for the visit based on ICD-9 diagnostic codes. Approximately 95% of physician-patient episodes of care in BC are reimbursed through the BC Ministry of Health fee-for-service.[2]

3) Medical Service Plan Consolidation File (01/1990 onwards) includes demographic information for each person registered with the provincial plan, such as age, sex, postal code (first three digits) used to determine rural vs. urban residence, neighborhood income quintile and local health area, and registration data.[3]

4) BC Vital Statistics (01/1996 onwards) - This data source includes date of death and primary cause of death (ICD-9 or ICD-10) from information provided on death certificates.[4]

5) Canadian Institute of Health Information (CIHI) Hospital Separation Abstracts (01/1990 onwards) - include up to 25 diagnostic codes per hospitalization using full 5-digit International Classification of Diseases, Version 9 (ICD-9) and/or ICD-10 codes, representing either the reason for admission or complications during hospitalization, hospital admission and discharge dates, and hospital transfers. One hundred percent of hospitalizations in BC are captured through this system.[5]

6) The National Ambulatory Care Reporting System (NACRS) (2012/2013 onwards) – is a tool for collecting data and reporting on all levels of ambulatory care within Canada including emergency departments (EDs), day surgery, and medical and surgical day clinics within hospitals, the community and private clinics.[6]

Acknowledgements:
All the data are made available via Population Data BC (https://www.popdata.bc.ca/). Access to data provided by the Data Stewards is subject to approval but can be requested for research projects through the Data Stewards or their designated service providers. The following data sets were used in this study: consolidation, hospital separations (DAD), MSP Practitioner File, NACRS, PharmaNet, and VS - deaths. You can find further information regarding these data sets by visiting the PopData project webpage at: https://my.popdata.bc.ca/project_listings/21-055/collection_approval_dates. All inferences, opinions, and conclusions drawn in this publication are those of the author(s), and do not reflect the opinions or policies of the Data Steward(s).

Supplementary Table S1. ICD-9 and ICD-10 codes for infection.

ICD-9	ICD-10
038 041 053 054 460 461 462 463 464 465 466 480 481 482 483 484 485 486 487 488 590 597 599 601.0 601.1 601.2 601.3 601.4 604 616.1 616.2 616.3 616.4 647 670 680 681 682 684 685 686 658.4 670.0 615.0 615.9 646.6 659.2 659.3 672 760.2 771 999.3 659.3 672 76.02 771 999.3	A40 A41 J00 J01 J02 J03 J04 J05 J06 J09 J10 J11 J12 J13 J14 J15 J16 J17 J18 J20 J21 J22 L00 L01 L02 L03 L04 L05 L06 L07 L08 N30 N34 N37 N39 N41.0 N41.3 N45 N71.0 N71.9 N76.0 N76.2 N77 O411 O85 O86 O23 O75.2 O75.3 P35 P36 P37 P38 P39

ICD-9
038 Septicemia
041 Bacterial infection in conditions classified elsewhere and of unspecified site
053 Herpes zoster
054 Herpes simplex
460 Acute nasopharyngitis
461 Acute sinusitis
462 Acute pharyngitis
463 Acute tonsillitis
464 Acute laryngitis and tracheitis
465 Acute upper respiratory infections of multiple or unspecified sites
466 Acute bronchitis and bronchiolitis
480 Viral pneumonia
481 Pneumococcal pneumonia
482 Other bacterial pneumonia
483 Pneumonia due to other specified organism
484 Pneumonia in infectious diseases classified elsewhere
485 Bronchopneumonia, organism unspecified
486 Pneumonia, organism unspecified
487 Influenza
488 Influenza due to identified avian influenza virus
590 Infections of kidney
597 Urethritis, not sexually transmitted, and urethral syndrome
599 Other disorders of urethra and urinary tract
601.0 Acute prostatitis
601.1 Chronic prostatitis
601.2 Abscess of prostate
601.3 Prostatocystitis
601.4 Prostatitis in diseases classified elsewhere
604 Orchitis and epididymitis
616.1 Vaginitis and vulvovaginitis
616.2 Cyst of Bartholin's gland
616.3 Abscess of Bartholin's gland
616.4 Other abscess of vulva
647 Infectious and parasitic conditions in the mother classifiable elsewhere, but complicating pregnancy, childbirth, or the puerperium
670 Major puerperal infection
680 Carbuncle and furuncle
681 Cellulitis and abscess of finger and toe
682 Other cellulitis and abscess
683 Acute lymphadenitis
684 Impetigo
685 Pilonidal cyst
686 Other local infections of skin and subcutaneous tissue

658.4 Infection of amniotic cavity
670 Major puerperal infection
615 Inflammatory diseases of uterus, except cervix
615.9 Unspecified inflammatory disease of uterus
646.6 Infections of genitourinary tract in pregnancy
659.2 Maternal pyrexia during labor, unspecified
659.3 Generalized infection during labor
672 Pyrexia of unknown origin during the puerperium
760.2 Maternal infections
771 Infections specific to the perinatal period
999.3 Other infection

ICD-10
A40 Streptococcal sepsis
A41 Other sepsis
J00 Acute nasopharyngitis [common cold]
J01 Acute sinusitis
J02 Acute pharyngitis
J03 Acute tonsillitis
J04 Acute laryngitis and tracheitis
J05 Acute obstructive laryngitis [croup] and epiglottitis
J06 Acute upper respiratory infections of multiple and unspecified sites
J09-J18 Influenza and pneumonia
J20-J22 Other acute lower respiratory infections
L00-L08 Infections of the skin and subcutaneous tissue
N30 Cystitis
N34 Urethritis and urethral syndrome
N37 Urethral disorders in diseases classified elsewhere
N39 Other disorders of urinary system
N41.0 Acute prostatitis
N41.3 Prostatocystitis
N45 Orchitis and epididymitis
N71.0 Acute inflammatory disease of uterus
N71.9 Inflammatory disease of uterus, unspecified
N76.0 Acute vaginitis
N76.2 Acute vulvitis
N77 Vulvovaginal ulceration and inflammation in diseases classified elsewhere
O41.1 Infection of amniotic sac and membranes
O85 Puerperal sepsis
O86 Other puerperal infections
O23 Infections of genitourinary tract in pregnancy
O75.2 Pyrexia during labour, not elsewhere classified
O75.3 Other infection during labour
P35 Congenital viral diseases
P36 Bacterial sepsis of newborn
P37 Other congenital infectious and parasitic diseases
P38 Omphalitis of newborn with or without mild haemorrhage
P39 Other infections specific to the perinatal period

Supplementary Appendix S2. The ITT and CACE models.

1. For the **ITT analysis**, the following quasi-Poisson model was used:

$$\frac{\mathrm{E}[y_i]}{Followup_i} = \exp(\beta_0 + f_1(Z_i, \beta_1) + \beta_2\, I_i + f_3(Z_i I_{i,}\, \beta_3)) \tag{1}$$

Where y_i is the outcome of interest (i.e., healthcare utilization outcome) for each individual i; $Followup_i$ represents the follow-up time for each individual *i* in person-years and is included in the model as an offset term; Z_i is our centered continuous running variable for individual's initiation time, representing the time (in days) from policy change date; I_i is an indicator variable indicating the pre-policy period or post-policy period ($I_i = 0$ if $Z_i < 0; I_i = 1$ if $Z_i \geq 0$). $\beta_0 + f_1(Z_i, \beta_1)$ and ($\beta_0 + f_1(Z_i, \beta_1) + \beta_2 + f_3(Z_i, \beta_3)$) represent separate curves of etanercept initiation time before and after policy change, respectively. The estimated coefficient $\hat{\beta}_2$ captures the discontinuity in the outcome at policy implementation and represents the immediate effect of the policy change on the outcome, and exponentiating $\hat{\beta}_2$ allow us to obtain the effect in rate ratio.

2. For the **CACE analysis**, the following two stage regression models were used:

$$y_{2,\mathrm{i}} = B\tilde{I}_i' + v_i \tag{2}$$

$$\frac{\mathrm{E}[y_i]}{Followup_i} = \exp\,(\beta_1 \mathrm{x}_i' + \beta_2 y_{2,i} + f_3(Z_i, \beta_3) + f_4(Z_i\, y_{2,i}, \beta_4) + v_i\rho + c_i) \tag{3}$$

First to control for the endogeneity, the endogenous variables $y_{2,i}$ (i.e., an indicator variable for whether a patient was treated by biosimilar [1] or originator [0]) is modeled in the first stage regression (Equation 2). Here $B\tilde{I}_i'$ represents the predicted value based on the augmented vector $\tilde{I}_i'$, which includes the baseline covariates x_i' and the instrumental variable I_i'; v_i is the residual from the regression which captures the unobserved confounding (estimated as $\hat{v}_i$).

Then, a second-stage quasi-Poisson model (Equation 3) was used to model the healthcare utilization outcome y_i. Here x_i' is a vector of exogenous covariates; $y_{2,i}$ is an endogenous regressor (i.e., biosimilar use in this study); $\beta_1 \mathrm{x}_i' + f_3(Z_i, \beta_3) + v_i\rho + c_i$ and $(\beta_1 \mathrm{x}_i' + \beta_2 + f_3(Z_i, \beta_3) + f_4(Z_i, \beta_4) + v_i\rho + c_i)$ represent separate curves of etanercept initiation time for originator and biosimilar users, respectively; v_i is the error term capturing unobserved confounding, and c_i is an independent disturbance term. The coefficients obtained from the second-stage regression were taken as the control function estimates[7]. The estimated coefficient $\hat{\beta}_2$ represents the effect of biosimilar use on the outcome locally around the time of policy implementation, and exponentiating $\hat{\beta}_2$ allow us to obtain the effect in rate ratio.

Supplementary Table S2. The intention-to-treat effect of policy implementation and complier average causal effect of biosimilar use on healthcare utilization outcomes via local linear regression.

Outcome	Parameter	Unadjusted effect estimates (95% CI) P-value	Adjusted [a] effect estimates (95% CI) P-value	Bandwidths [b]
PV rate	ITT	2·73 (-8·98–14·43) 0·65	-0·68 (-11·87–10·51) 0·91	497
	CACE	2·03 (-27·30–31·36) 0·65	-2·50 (-43·44–38·45) 0·91	497
ACH rate	ITT	-0·03 (-0·30, 0·24) 0·81	-0·06 (-0·33–0·20) 0·65	746
	CACE	-0·10 (-0·86–0·67) 0·81	-0·18 (-0·93–0·58) 0·65	746
IRH rate	ITT	0·01 (-0·01–0·03) 0·41	0·01 (-0·01–0·03) 0·60	636
	CACE	0·03 (-0·04–0·09) 0·41	0·02 (-0·05–0·08) 0·60	636
LOS	ITT	0·04 (-1·00–1·07) 0·95	-0·09 (-1·10–0·93) 0·87	797
	CACE	0·10 (-2·80–3·01) 0·95	-0·24 (-3·12–2·64) 0·87	797
ERV rate	ITT	-0·06 (-0·49–0·37) 0·79	-0·09 (-0·52–0·33) 0·67	514
	CACE	-0·21 (-1·69–1·28) 0·79	-0·33 (-1·83–1·17) 0·67	514

* Estimates obtained from local linear regression with optimal bandwidths around the cut-off obtained via Imbens and Kalyanaraman bandwidths selector.[8] Bandwidths represent days or distance from July 18, 2017. Rates were defined as number of outcomes per 1 follow-up year. Effect estimates were in rate differences. Abbreviations: ACH: All-cause hospitalization; CACE: complier average causal effect; CI: Confidence interval; ERV: emergency room visit; IRH: infection-related hospitalization; ITT: intention-to-treat; LOS: length of hospital stays; PV: physician visit.

[a] Adjusted baseline covariates include sociodemographic characteristics, comorbidities, and medication use.

[b] Bandwidths used for estimating both the unadjusted and adjusted effects.

Supplementary Table S3. The intention-to-treat effect of policy implementation on healthcare utilization outcomes with MSE-optimal bandwidths via quasi-Poisson Regression.

Outcome	Parameter	Pre-policy (95% CI)	Post-policy (95% CI)	Unadjusted effect estimates	Adjusted [a] effect estimates	Bandwidths [b]	Bandwidths [c]
PV rate	ITT	57·68 (50·3–66·15)	63·06 (54·97–72·35)	1·09 (0·90–1·33) 0·37	0·99 (0·84–1·18) 0·93	447	435
ACH rate	ITT	0·30 (0·14–0·62)	0·33 (0·18–0·60)	1·12 (0·43–2·91) 0·81	0·89 (0·37–2·13) 0·79	331	333
IRH rate	ITT	0·01 (0·002–0·04)	0·01 (0·002–0·04)	1·09 (0·12–10·10) 0·94	0·69 (0·05–10·02) 0·78	258	260
LOS	ITT	0·85 (0·34–2·09)	0·90 (0·43–1·89)	1·07 (0·33–3·42) 0·91	0·73 (0·30–1·77) 0·49	483	486
ERV rate	ITT	0·51 (0·31–0·85)	0·52 (0·29–0·95)	1·02 (0·47–2·23) 0·96	0·86 (0·40–1·87) 0·71	354	355

* Estimates obtained from Quasi-Poisson regression with optimal bandwidths around the cut-off obtained via the mean square error (MSE)-optimal bandwidths algorithm.[9] Bandwidths represent days or distance from July 18, 2017. Rates were defined as number of outcomes per 1 follow-up year. ITT effect estimates were in rate ratios. Confidence intervals (CIs) obtained using critical value = 1·96. Abbreviations: ACH: All-cause hospitalization; CACE: complier average causal effect; CI: Confidence interval; ERV: emergency room visit; IRH: infection-related hospitalization; ITT: intention-to-treat; LOS: length of hospital stays; PV: physician visit.

[a] Adjusted baseline covariates include sociodemographic characteristics, comorbidities, and medication use.

[b] Bandwidths for estimating the unadjusted effect.

[c] Bandwidths for estimating the adjusted effect.

Supplementary Table S4. The intention-to-treat effect of policy implementation and complier average causal effect of biosimilar use on healthcare utilization outcomes with MSE-optimal bandwidths via local linear regression.

Outcome	Parameter	Unadjusted effect estimates (95% CI) P-value	Adjusted [a] effect estimates (95% CI) P-value	Bandwidths [b]	Bandwidths [c]
PV rate	ITT	2·45 (-9·90–14·8) 0·70	-1·41 (-13·40–10·58) 0·82	447	435
	CACE	6·78 (-46·69–60·26) 0·80	-2·50 (-45·81–40·82) 0·91	410	476
ACH rate	ITT	0·06 (-0·25–0·37) 0·70	0·02 (-0·29–0·32) 0·92	331	333
	CACE	0·28 (-1·01–1·56) 0·67	0·08 (-1·26–1·43) 0·90	386	372
IRH rate	ITT	<0·01 (-0·02–0·02) 0·71	<0·01 (-0·02–0·02) 0·95	258	260
	CACE	0·02 (-0·10–0·15) 0·70	<0·01 (-0·11–0·12) 0·94	255	299
LOS	ITT	-0·17 (-1·45–1·10) 0·79	-0·39 (-1·64–0·86) 0·54	483	486
	CACE	-0·66 (-5·36–4·04) 0·78	-1·67 (-6·51–3·17) 0·50	479	468
ERV rate	ITT	0·04 (-0·40–0·48) 0·86	0·01 (-0·45–0·47) 0·97	354	355
	CACE	0·18 (-1·88–2·23) 0·87	0·02 (-2·21–2·25) 0·98	357	341

* Estimates obtained from local linear regression with optimal bandwidths around the cut-off obtained via the mean square error (MSE)-optimal bandwidths algorithm.[9] Bandwidths represent days or distance from July 18, 2017. Rates were defined as number of outcomes per 1 follow-up year. Effect estimates were in rate differences. Abbreviations: ACH: All-cause hospitalization; CACE: complier average causal effect; CI: Confidence interval; ERV: emergency room visit; IRH: infection-related hospitalization; ITT: intention-to-treat; LOS: length of hospital stays; PV: physician visit.

[a] Adjusted baseline covariates include sociodemographic characteristics, comorbidities, and medication use.

[b] Bandwidths for estimating the unadjusted effect.

[c] Bandwidths for estimating the adjusted effect.

Supplementary Table S5. The intention-to-treat effect of policy implementation on healthcare utilization outcomes among adalimumab initiators: A robustness check.

Outcome	Parameter	Pre-policy (95% CI)	Post-policy (95% CI)	Unadjusted effect estimates (95% CI) P-value	Adjusted [a] effect estimates (95% CI) P-value	Bandwidths [b]
PV rate	ITT	51·84 (46·36, 57·96)	57·90 (52·33, 64·06)	1·12 (0·96, 1·30) 0·15	1·10 (0·97, 1·26) 0·15	530
ACH rate	ITT	0·40 (0·26, 0·61)	0·35 (0·25, 0·50)	0·87 (0·50, 1·51) 0·63	0·91 (0·52, 1·58) 0·74	623
IRH rate	ITT	0·02 (0·01, 0·06)	0·02 (0·01, 0·06)	1·01 (0·28, 3·58) 0·99	0·92 (0·27, 3·14) 0·89	635
LOS	ITT	1·06 (0·66, 1·71)	1·20 (0·71, 2·01)	1·13 (0·56, 2·29) 0·73	1·03 (0·55, 1·90) 0·93	784
ERV rate	ITT	0·58 (0·40, 0·84)	0·58 (0·39, 0·88)	1·01 (0·57, 1·76) 0·99	0·99 (0·57, 1·72) 0·96	475

Estimates obtained from Quasi-Poisson regression with optimal bandwidths around the cut-off obtained via Imbens and Kalyanaraman bandwidths selector.[8] Bandwidths represent days or distance from July 18, 2017. Rates were defined as number of outcomes per 1 follow-up year. ITT effect estimates were in rate ratios. Confidence intervals (CIs) obtained using critical value = 1·96. Abbreviations: ACH=all-cause hospitalization; CI=Confidence interval; ERV=emergency room visit; IRH=infection-related hospitalization; ITT=intention-to-treat; LOS=length of hospital stays; PV=physician visit.

[a] Adjusted baseline covariates include sociodemographic characteristics, comorbidities, and medication use.

[b] Bandwidths used for estimating both the unadjusted and adjusted effects.

Supplementary Table S6. The intention-to-treat effect of policy implementation on healthcare utilization outcomes excluding data overlapping with the COVID-19 period.

Outcome	Parameter	Pre-policy (95% CI)	Post-policy (95% CI)	Unadjusted effect estimates (95% CI) P-value	Adjusted [a] effect estimates (95% CI) P-value	Bandwidths [b]
PV rate	ITT	59·64 (52·21, 68·12)	64·09 (55·79, 73·63)	1·08 (0·89, 1·30) 0·46	1·00 (0·85, 1·18) 0·99	463
ACH rate	ITT	0·34 (0·18, 0·63)	0·33 (0·20, 0·54)	0·97 (0·44, 2·15) 0·95	0·91 (0·43, 1·94) 0·81	528
IRH rate	ITT	0·01 (0·002, 0·03)	0·01 (0·01, 0·04)	1·63 (0·33, 7·94) 0·55	1·04 (0·19, 5·65) 0·97	509
LOS	ITT	0·60 (0·28, 1·32)	0·59 (0·28, 1·27)	0·98 (0·33, 2·93) 0·98	0·82 (0·30, 2·25) 0·70	510
ERV rate	ITT	0·47 (0·30, 0·75)	0·47 (0·28, 0·79)	1·00 (0·50, 1·99) 0·99	1·01 (0·51, 1·98) 0·98	551

Estimates obtained from Quasi-Poisson regression with optimal bandwidths around the cut-off obtained via Imbens and Kalyanaraman bandwidths selector.[8] Bandwidths represent days or distance from July 18, 2017. Incident etanercept users initiating after March 1, 2019 were excluded from the analysis. Patients were followed for 1-year to capture healthcare utilization outcomes so that follow up period is the same for all patients and has no overlap with the COVID pandemic. Rates were defined as number of outcomes per 1 follow-up year. ITT effect estimates were in rate ratios. Confidence intervals (CIs) obtained using critical value = 1·96. Abbreviations: ACH=all-cause hospitalization; CI=Confidence interval; ERV=emergency room visit; IRH=infection-related hospitalization; ITT=intention-to-treat; LOS=length of hospital stays; PV=physician visit.

[a] Adjusted baseline covariates include sociodemographic characteristics, comorbidities, and medication use.

[b] Bandwidths used for estimating both the unadjusted and adjusted effects.

Supplementary Table S7. The complier average causal effect of biosimilar use on healthcare utilization outcomes excluding data overlapping with the COVID-19 period.

Outcome	Parameter	Unadjusted effect estimates (95% CI) P-value	Adjusted [a] effect estimates (95% CI) P-value
**PV rate**	CACE	0·95 (0·76, 1·20)<br>0·67	0·83 (0·67, 1·04)<br>0·11
**ACH Rate**	CACE	0·99 (0·56, 1·76)<br>0·99	1·05 (0·56, 1·97)<br>0·89
**IRH rate**	CACE	1·01 (0·08, 12·98)<br>0·99	Not Converged [b]
**LOS**	CACE	1·11 (0·40, 3·12)<br>0·84	0·99 (0·35, 2·82)<br>0·99
**ERV rate**	CACE	0·98 (0·38, 2·53)<br>0·98	0·96 (0·38, 2·41)<br>0·93

Estimates obtained using Quasi-Poisson regression with instrumental variable (IV) control function method. Incident etanercept users initiating after March 1, 2019 were excluded from the analysis. Patients were followed for 1-year to capture healthcare utilization outcomes so that follow up period is the same for all patients and has no overlap with the COVID pandemic. Rates were defined as number of outcomes per 1 follow-up year. CACE estimates were in rate ratios. Confidence intervals (CIs) obtained using critical value = 1·96. Abbreviations: ACH=all-cause hospitalization; CACE=complier average causal effect; CI=Confidence interval; ERV=emergency room visit; IRH=infection-related hospitalization; LOS=length of hospital stays; PV=physician visit.

[a] Adjusted baseline covariates include sociodemographic characteristics, comorbidities, and medication use.

[b] The adjusted CACE effect for infection-related hospitalization outcome did not converge because of insufficient sample size and events occurred among biosimilar users in the post-policy change period (due to the exclusion of patients initiating after March 1, 2019, and the short one-year follow-up period).

Supplementary Appendix S3. Assessing underlying assumptions in our ITT Analysis (A sharp RD design).

The main underlying assumption in obtaining the effect of policy implementation on healthcare utilization was called the *exchangeability* assumption (or the *continuity* assumption).[10,11] The validity of the RD design depends closely on this assumption that there is a locally randomized experiment around the cut-off point. In other words, individuals immediately below the cut-off share the same distribution of both measured and unmeasured baseline characteristics as those individuals immediately above the cut-off. Under the potential-outcomes framework of causal inference,[12,13] there exists a pair of potential outcomes — $Y_i(1)$ represents what would happen if an individual was exposed to the treatment and $Y_i(0)$ represents what would happen if the same individual was not exposed to the treatment. Then, the difference $[Y_i(1)$ - $Y_i(0)]$ is the causal effect of the treatment for that particular individual. The exchangeability assumption in RD design assumes the treatment assignment is independent of the potential outcomes and individuals close to the treatment cut-off is exchangeable. This enables us to use the average outcome of individuals immediate below the treatment cut-off (who are not exposed to the biosimilar policy) as a valid counterfactual for those right above the cutoff (who are not exposed to the biosimilar policy).

The exchangeability assumption cannot be fully verified (i.e., not fully testable). In our study setting, the assumption is highly plausible, because the cut-off of treatment eligibility is based on the policy change date. "Manipulation" is less likely than preference-based cut-off such as physician's prescription behavior. The PharmaCare in BC provides universal coverage for biologics under the special authority for arthritis patients. Patients still have some out-of-pocket costs due to a deductible, which is proportional to their income. Both originators and biosimilars exceed the deductible threshold, so prescribing the less expensive biosimilar does not reduce the patient's out-of-pocket cost. Thus, it is unlikely that patients would be refrained from being prescribed the medication until after the policy date. Although we do not anticipate patients altering/manipulating their drug prescription dates in response to the policy change, it remains crucial to partially test for the assumption.

We assessed for violations as follows:

1) We formally performed a density test (the Cattaneo, Jansson, and Ma test[14,15]) to examine the continuity of the density of the running variable at the cut-off. A jump in the density (i.e., discontinuity) can serve as evidence for sorting around the cut-off point,[16] suggesting that allocation around the cut-off is not random. Our results indicate that there is no sorting of patients around the cut-off point. The distribution of our running variable (i.e., patient's biologics initiation time) is continuous and unaffected by the cut-off (density test t statistics = 0·65, p=0·52) (**Supplementary Figure S1**).
2) We formally performed a covariates balance test. We ran the RD analysis in the same specifications as the main regression discontinuity analyses on all the measured characteristics as outcomes to test for any discontinuity. We found no discontinuity around the cut-off (see **Supplementary Figures S2-4**), suggesting that patients' (observed) characteristics were similar, which increases our confidence of a locally randomized experiment around the cut-off. While unlikely, it is still important to acknowledge that this does not completely rule out the potential existence of imbalances in unmeasured covariates that might be related to the risk of the outcome.

Supplementary Appendix S4. Assessing underlying assumptions in our CACE Analysis (A fuzzy RD design with IV analysis).

In our fuzzy RD with IV analysis, we estimated a complier average causal effect (CACE), also known as local average treatment effect (LATE). **Supplementary Figure S5** shows a causal diagram demonstrating our fuzzy RD with IV estimation. The instrument variable I represents a binary treatment assignment variable, indicating whether a patient started etanercept before [0] or after [1] cut-off (i.e., whether or not exposed to policy change). Treatment condition Y_2 is a binary variable indicating the type of biologic treatment used, whether originator etanercept [0] or biosimilar etanercept [1]. Outcome Y is healthcare utilization rate for each patient (i.e., number of physician visits per 1 follow-up year).

We first (1) regress the treatment Y_2 on the instrumental variable I and baseline covariates; and then (2) the second-stage quasi-Poisson model included two separate curves of the etanercept initiation time (measured as days from the policy change) for originator (Y_2=0) and biosimilar incident users (Y_2=1), respectively, using penalized splines, an indicator variable for biosimilar incident users (Y_2) to capture the outcome difference for biosimilar vs originator use at policy implementation, baseline covariates, and the residual of the first-stage regression as the control function to control for unobserved confounders of biosimilar use. The coefficients of the second-stage regression are taken as the control function estimates.

There are four underlying assumptions involved in fuzzy RD with IV[17,18]:

1. The *relevance* assumption: The instrumental variable I is associated with the treatment Y_2 ("relevance").

2. The *exchangeability* assumption: I does not share common causes with the outcome Y (no unmeasured confounders).

3. The *exclusion restriction* assumption: I affects the outcome Y only through Y_2 (No direct effect on the outcome beyond its effect on the treatment).

4. The *monotonicity* assumption: There are no units who receive a treatment condition Y_2 that is always opposite to their treatment assignment I ("No defiers").

Assessing Assumption 1: Relevance

(1) In our fuzzy RD with IV analysis, our instrumental variable was exposure to the biosimilar policy (which was defined based on the policy change date). Hence, one way to prove IV relevance is to show a discontinuity in the probability of biosimilar initiation at the biosimilar policy change date. Etanercept new users were grouped into quarterly cohorts and the probability of biosimilar use for each cohort was calculated (i.e., shown as the red dots in figure 1 in the main text). We can visually see a discontinuity at the policy change date. The probability immediately before and after cut-off was 0·047 and 0·439, respectively. We then formally performed an RD analysis on initiation time vs probability of biosimilar use as outcome with cut-off set at the policy change date. Difference in probability of biosimilar use (i.e., risk difference) at the cut-off was estimated to be 0·50 (95% CI 0·36, 0·64).
(2) We performed a partial F test, comparing the full model including the IV (i.e., exposure to the biosimilar policy) against the simplified model without the IV to predict biosimilar use. The results strongly suggest that the instrument is associated with the treatment with a partial F-statistic of 370·1 (p<0·0001)).

Assessing Assumption 2: Exchangeability

Theoretically, it is not possible to empirically verify the exchangeability assumption. However, we have addressed potential violations of this assumption through the application of density tests and covariate balance tests within the RD framework (See **Supplementary Figures S2-4**). These tests serve to assess the comparability of people immediately above and below the cut-off. Furthermore, we have carefully reviewed the timing of policy

implementations and found no concurrent policies other than the biosimilar policy that would be expected to affect healthcare utilization.

Assessing Assumption 3: Exclusion Restriction

The exclusion restriction assumption also cannot be empirically verified using our data. In our setting, we do not anticipate that the biosimilar policy change has any direct effect on healthcare utilization outcomes other than through its effect via the biosimilar treatment. We believe these assumptions are valid, recognizing the multifaceted nature of healthcare utilization, which is influenced by a complex interplay of systemic, provider-related, and individual-level factors. This complexity implies that any direct effect of the biosimilar policy change, if present, bypassing the biosimilar treatment, would be difficult to manifest immediately and systematically across the board at the policy implementation date.

Assessing Assumption 4: Monotonicity

Under the monotonicity assumption, there must be no patients who would have always taken up treatment if ineligible and would also have always not taken up treatment if eligible (i.e., no defiers in the population). This assumption is not empirically verifiable. In our study setting, the likelihood of encountering defiers is minimal, given that our instrumental variable is the exposure to the biosimilar policy, which is determined by the date of policy change. A policy change provides a clear-cut criterion that applies uniformly to the population affected by the policy, making it a more reliable instrument for the purposes of this study than physician preference-based instruments. Furthermore, Table 1 shows that only a small number (9 out of 1616=0·6%) of people took biosimilar before policy change. Because defiers are a subset of these non-compliers (i.e., people who took biosimilar before policy change), this means the proportion of defiers cannot exceed 0·6%. Thus, even in the event of the assumption being violated, its impact on our results is expected to be negligible.

Supplementary Figure S1. Density plot of the running variable.

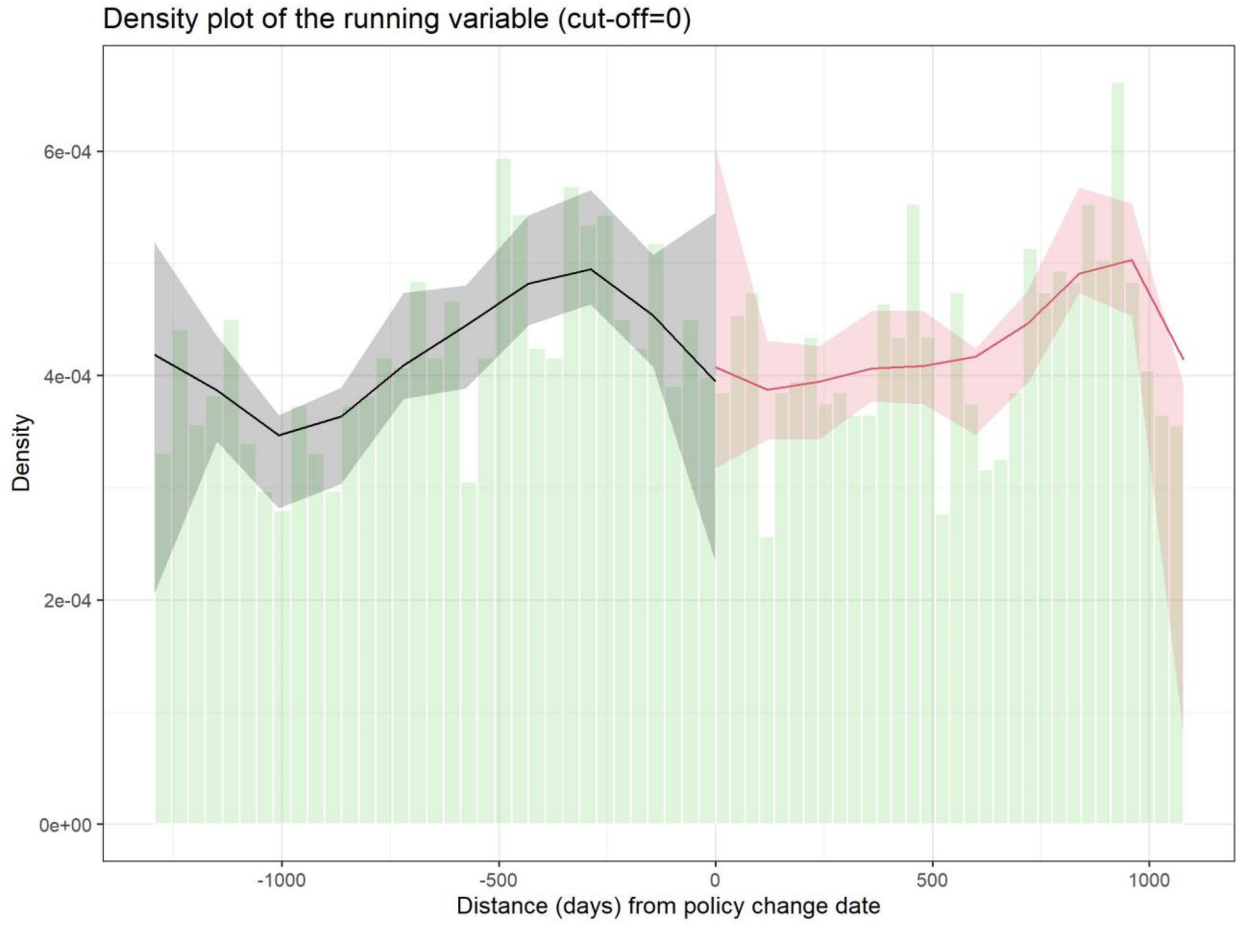

Supplementary Figure S1. Density plot of the running variable (assessed with distance/days from policy change date).

Supplementary Figure S2. Covariates balance check (demographics).

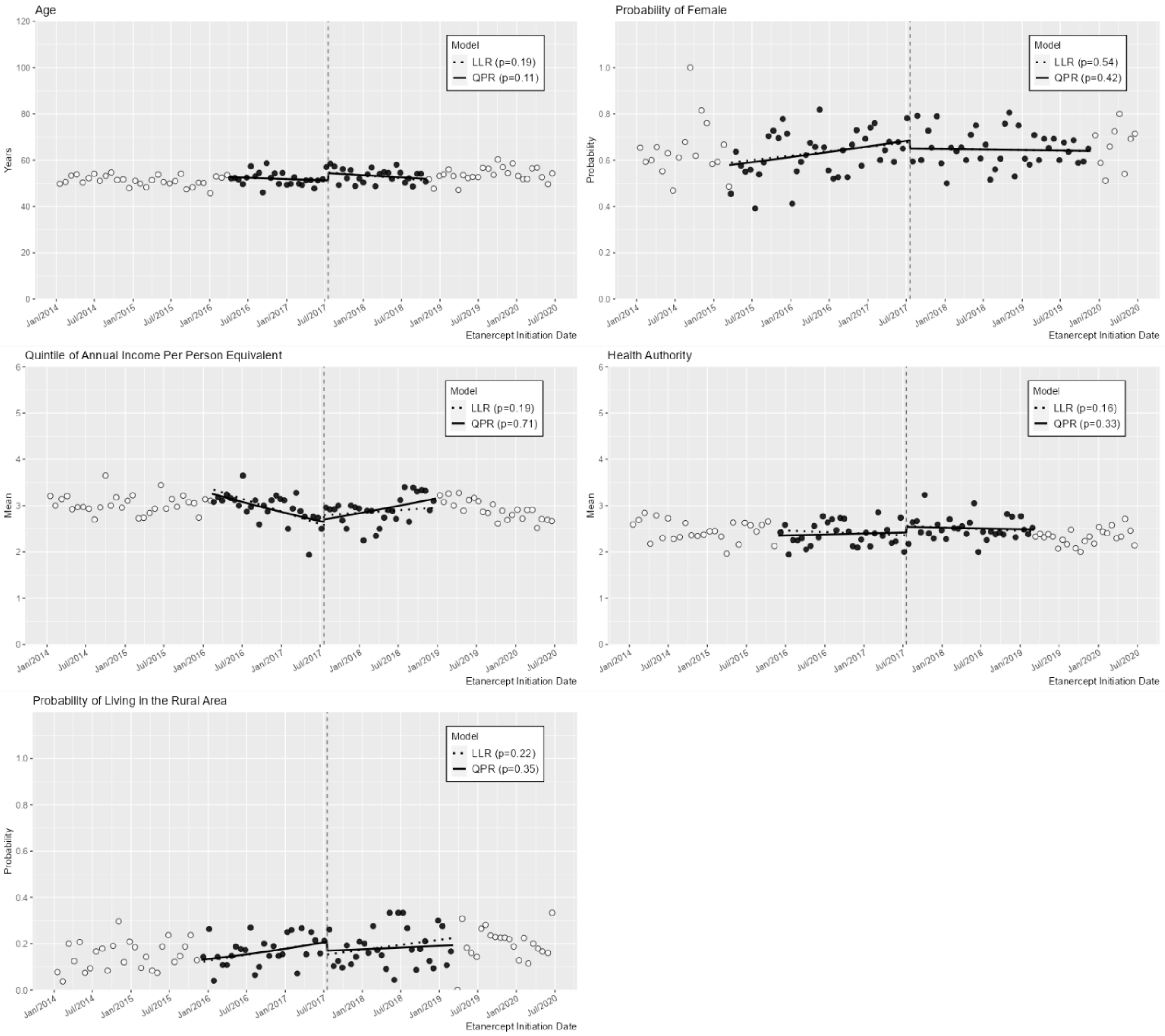

Supplementary Figure S2. Covariates balance check (demographics). P-values indicate the statistical significance for testing the null hypothesis for absence of any discontinuities at the cut-off for each demographic variable, obtained either through quasi-Poisson regression (QPR) or local linear regression (LLR).

Supplementary Figure S3. Covariates balance check (comorbidities).

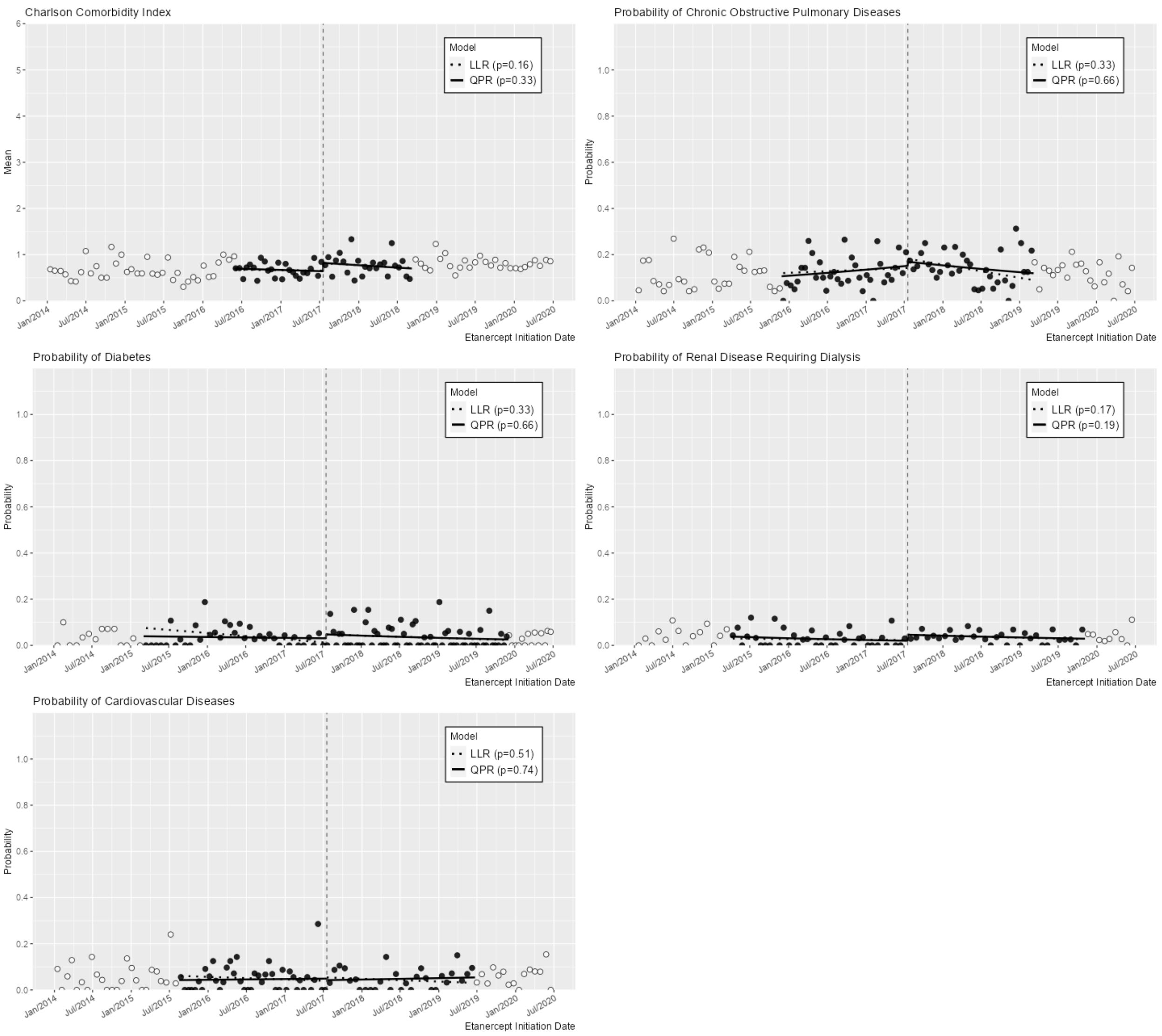

Supplementary Figure S3. Covariates balance check (comorbidities). P-values indicate the statistical significance for testing the null hypothesis for absence of any discontinuities at the cut-off for each comorbidity variable, obtained either through quasi-Poisson regression (QPR) or local linear regression (LLR).

Supplementary Figure S4. Covariates balance check (medication use).

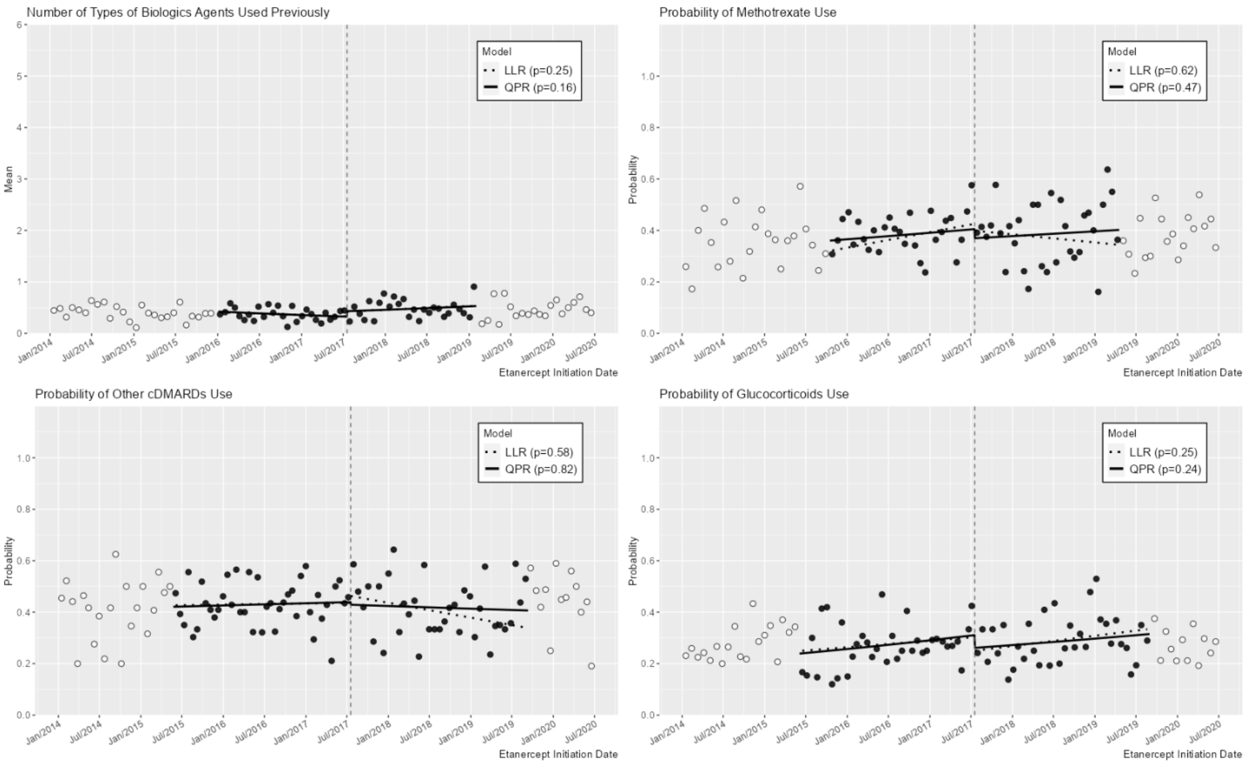

Supplementary Figure S4. Covariates balance check (medication use). P-values indicate the statistical significance for testing the null hypothesis for absence of any discontinuities at the cut-off for each medication use variable, obtained either through quasi-Poisson regression (QPR) or local linear regression (LLR). cDMARDs: conventional disease-modifying anti-rheumatic drugs.

Supplementary Figure S5. A causal diagram demonstrating our fuzzy RD with IV estimation.

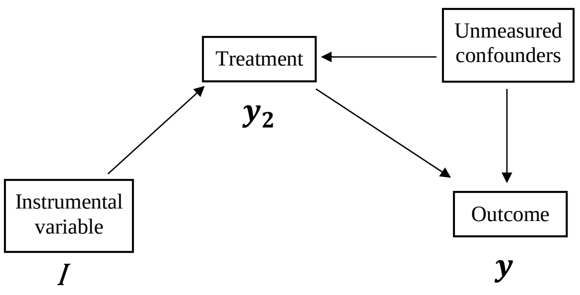

References

1 British Columbia Ministry of Health [creator] (2022). PharmaNet. V2. BC Ministry of Health [publisher]. Data Extract. British Columbia Ministry of Health (2022). Available from: http://www.popdata.bc.ca/data.

2 British Columbia Ministry of Health [creator] (2022). Medical Service Plan (MSP) Payment Information File. V2. Population Data BC [publisher]. Data Extract. British Columbia Ministry of Health (2022). Available from: http://www.popdata.bc.ca/data.

3 British Columbia Ministry of Health [creator] (2022): Consolidation File (MSP Registration & Premium Billing). V2. Population Data BC [publisher]. Data Extract. British Columbia Ministry of Health (2022). Available from: http://www.popdata.bc.ca/data.

4 BC Vital Statistics Agency [creator] (2022). Vital Statistics Death. V2. Population Data BC [publisher]. Data Extract BC Vital Statistics Agency (2022). Available from: http://www.popdata.bc.ca/data.

5 Canadian Institute for Health Information [creator] (2022). Discharge Abstract Database (Hospital Separations). V2. Population Data BC [publisher]. Data Extract. Canadian Institute for Health Information (2022). Available from: http://www.popdata.bc.ca/data.

6 British Columbia Ministry of Health [creator] (2022): The National Ambulatory Care Reporting System (NACRS). V2. Population Data BC [publisher]. Data Extract. British Columbia Ministry of Health (2022). Available from: http://www.popdata.bc.ca/data.

7 Guo Z, Small DS. Control Function Instrumental Variable Estimation of Nonlinear Causal Effect Models. *Journal of Machine Learning Research* 2016; **17**: 1–35.

8 Imbens G, Kalyanaraman K. Optimal Bandwidth Choice for the Regression Discontinuity Estimator. The Review of Economic Studies 2012; 79: 933–59.

9 Calonico S, Cattaneo MD, Titiunik R. Robust Nonparametric Confidence Intervals for Regression-Discontinuity Designs. Econometrica 2014; 82: 2295–326.

10 Lee DS, Lemieux T. Regression Discontinuity Designs in Economics. Journal of Economic Literature 2010; 48: 281–355.

11 Oldenburg CE, Moscoe E, Bärnighausen T. Regression Discontinuity for Causal Effect Estimation in Epidemiology. Curr Epidemiol Rep 2016; 3: 233–41.

12 Rubin DB. Estimating causal effects of treatments in randomized and nonrandomized studies. Journal of Educational Psychology 1974; 66: 688–701.

13 Imbens GW, Rubin DB. Causal Inference for Statistics, Social, and Biomedical Sciences: An Introduction. Cambridge: Cambridge University Press, 2015 DOI:10.1017/CBO9781139025751.

14 Cattaneo MD, Titiunik R. Regression Discontinuity Designs. Annual Review of Economics 2022; 14: 821–51.

15 Cattaneo MD, Jansson M, Ma X. Local regression distribution estimators. Journal of Econometrics 2024; 240: 105074.

16 McCrary J. Manipulation of the running variable in the regression discontinuity design: A density test. Journal of Econometrics 2008; 142: 698–714.

17 Swanson SA, Hernán MA. Commentary: how to report instrumental variable analyses (suggestions welcome). Epidemiology 2013; 24: 370–4.

18 Didelez V, Meng S, Sheehan NA. Assumptions of IV Methods for Observational Epidemiology. Statistical Science 2010; 25: 22–40.
